# Haemozoin enhances MMP-9 production through MAPK p38-dependent mechanisms in human adherent monocytes

**DOI:** 10.1186/1475-2875-13-S1-P73

**Published:** 2014-09-22

**Authors:** Amina Khadjavi, Elena Valente, Giuliana Giribaldi, Mauro Prato

**Affiliations:** 1 Dipartimento di Neuroscienze, Università di Torino, Torino, Italy; 2 Dipartimento di Oncologia, Università di Torino, Torino, Italy

## 

In human adherent monocytes matrix metalloproteinase-9 (MMP-9) expression and secretion is upregulated by the lipid moiety of natural haemozoin (nHz, malarial pigment). A role for 15-(S, R)-hydroxy-6, 8, 11, 13-eicosatetraenoic acid (15-HETE), a nHZ lipoperoxidation product, has been suggested. Here, the underlying mechanisms were investigated, focusing on the involvement of mitogen-activated protein kinases (MAPKs). Either early or late p38 MAPK phosphorylation was induced by nHz, which did not modify basal phosphorylation and expression ratios of extracellular signal-regulated kinase-1/2 and c-jun N-terminal kinase-1/2. 15-HETE mimicked nHZ effects on p38 MAPK. Lipid-free synthetic (s)HZ and delipidized (d)HZ did not. Both nHZ and 15-HETE promoted the phosphorylation of MAPK-activated protein kinase-2, a known substrate of p38 MAPK. Such an effect was abolished by SB203580 (synthetic p38 MAPK inhibitor). SB203580 also abrogated the nHZ-dependent and 15-HETE-dependent enhancement of MMP-9 mRNA and protein levels in cell lysates and supernatants. These data suggest that nHZ and 15-HETE upregulate MMP-9 expression and secretion through the activation of p38 MAPK pathway in human adherent monocytes. This work provides new evidence on the mechanisms underlying MMP-9 deregulation in malaria. These data might help to design new specific drugs for adjuvant therapy in complicated malaria.

